# Canopy hyperspectral characteristics and yield estimation of winter wheat (*Triticum aestivum*) under low temperature injury

**DOI:** 10.1038/s41598-019-57100-8

**Published:** 2020-01-14

**Authors:** Yongkai Xie, Chao Wang, Wude Yang, Meichen Feng, Xingxing Qiao, Jinyao Song

**Affiliations:** Institute of Dry Farming Engineering, Shanxi Agricultural University, Taigu, 030801 Shanxi China

**Keywords:** Climate and Earth system modelling, Climate and Earth system modelling, Environmental impact, Environmental impact

## Abstract

To evaluate the effect of low-temperature stress in winter wheat during the early growth stages, the response regularity of the canopy spectral reflectance was evaluated. Besides, winter wheat yield during the maturation stage and the relationship between yield and canopy spectral reflectance were also analyzed. Two multivariate methods, namely, the successive projections algorithm (SPA) and multiple linear regression (MLR), were combined to explore the relationship between the spectral reflectance and yield. Our results showed that the green peak and red valley in visible wavelengths altered obviously and the red edge gradually moved towards blue wavebands. The canopy spectral reflectance in the near-infrared wavebands increased with an increase in low-temperature stress intensity. Moreover, the reflectance proved that the red edge region under low-temperature stress is related to winter wheat yield, and approximately 38% of extracted wavebands were concentrated in the red edge region (680–780 nm). Compared with the predictive MLR models, the model calibrated during the flowering period of winter wheat (25 days post low-temperature treatment) had better performance in predicting crop yield. Whole-spectrum predictive models based on the principle component regression (PCR) method and Normalized Difference Vegetation Index (NDVI) models based on MLR were also established. Moreover, the performance of three kinds of calibration methods and the validation result of the field test were compared to select the optimal monitoring stage and technique to estimate the yield in the early growth stage of winter wheat under low-temperature stress. This study could provide a theoretical basis and practical reference for hyperspectral assessment of yield in winter wheat during low-temperature stress.

## Introduction

In recent years, frequent extreme weather events have seriously affected crop production. In particular, low temperature has reduced wheat production levels^1–5^. In northern China, late spring cold mainly occurs during the jointing stage of winter wheat, which has become one of the most common natural disasters that restricts the formation and quality of winter wheat yield^6^. After low-temperature injury, real-time, rapid and accurate assessment of the area and degree of winter wheat injury are of great significance for helping government to formulate relevant policies. The traditional field survey method is time-consuming, labourious and time-limited^7^. Hyperspectral remote sensing data have more bands and higher spectral resolution ratios and can provide widely significant spectral information. Therefore, spectral remote sensing technology provides an effective technical means to address this issue^8–10^.

In terms of macroscopic remote sensing, Zhang *et al*. used the WOFOST crop model in combination with meteorological data and winter wheat observation data in Shangqiu city since 1980 to 2005 to simulate the five-year frost disaster^11^. Zhong *et al*. used observation data of two decades from 36 agricultural meteorological observatories in the Huanghuai wheat region and its surrounding areas to analyse the main factors affecting the jointing period of winter wheat and established an equation for the jointing rate change over time^12^. Feng *et al*. used the NDVI obtained with MODIS data to analyse the occurrence, distribution and severity of winter wheat frost disasters in Shanxi Province, which were combined with meteorological observation data and field survey data^13^. National and international researchers conducted a series of studies on the frost damage of winter wheat, with some promising results. However, the precision of macroscopic remote sensing research is not very high, and the accuracy of monitoring of freeze stress in winter wheat under macroscopic conditions needs to be improved. This low accuracy may be due to freeze stress, unclear mechanisms of spectral information and inaccurate extraction of spectral feature information^14^. Poor performance may be observed when a crop model is applied in a large area because of high uncertainties in the spatial distributions of meteorological forcings, soil properties, initial model conditions, crop parameters and field management practices, which results in biased simulations^15^. Therefore, hyperspectral reflectance technology may provide tools to overcome the above problems in winter wheat under the influence of low-temperature stress. In a study on changes in physiological and ecological parameters after freeze stress, Li *et al*. used remote sensing to monitor winter wheat freeze damage through the relationships between low-temperature stress and spectral characteristics and between physiological and ecological parameters. Frost damage caused the loss of grain yield^16^. Ren *et al*. explored the sensitive response of winter wheat canopy spectra under frost damage by using hyperspectral reflectance technology^17^. Due to the popularity of hyperspectral technology in monitoring crops after freeze stress, many scholars have made a series of changes to the original spectral data. Wang Huifang established a model of the severity of freeze stress based on principal component analysis, which could effectively and rapidly reflect the severity of freeze stress in winter wheat^18^. Duan *et al*. studied the spectral characteristics of wheat before and after frost damage and the sensitive band for remote sensing diagnosis and extracted the coverage characteristics of wheat before and after freeze stress by image processing techniques^19^. Li *et al*. carried out inverse logarithm, first derivative, and second derivative transformations of the original hyperspectral data, which were correlated with chlorophyll content, and found the eigenvalues describing freeze stress to obtain bands and indexes that could be used to assess and evaluate the degree of frost damage^20^. Wu *et al*. employed wave analysis and many features of red edge parameters to monitor the effects of late frost on early stages of winter wheat by several methods, including the sensitivity and stability of correlation analysis and linear regression modelling^21^. Meng Lei explored the forecasting method of winter wheat yield variation with superimposed effects of soil surface moisture and nighttime frost damage by correlation analysis and one-dimensional linear regression to fit yield factors and spectral characteristics of frozen winter wheat under different soil moisture levels^22^. Shi *et al*. conducted correlation analysis of 15 spectral characteristic parameters, including the position and amplitude of the red edge and 4 plant height factors, and established a stepwise regression model^23^. Feng *et al*. established a model for the FICEI of winter wheat, and seven sensitive bands (574 nm, 702 nm, 1093 nm, 1111 nm, 1210 nm, 1302 nm, and 1333 nm) were extracted according to variable importance analysis^24^. After the occurrence of low-temperature injury in winter wheat, Katherine used non-destructive indirect selection of wheat genotypes with improved nitrogen use traits and canopy spectroscopy to improve the nitrogen use efficiency of wheat and reduce the need for nitrogen fertilizers^25^. Shiferaw studied the physiology and adaptation of winter wheat to soil moisture changes in the Pacific Northwest and highlighted the use of spectro-radiometric measurements to measure the grain yield^26^. Then, Shiferaw indirectly measured the yield potential and stability of winter wheat in the Pacific Northwest by spectral reflectance^27^. At present, many researchers apply spectral technology in real-time monitoring and production estimation^28–33^. However, hyperspectral data such as AVIRIS, OMIS, CHRIS, and Hyperion data, among others, have limitations, such as a high price, narrow width, and long return cycle^34^. However, very few studies have focused on applying spectral techniques in the real-time monitoring of winter wheat to extract characteristic variables under freeze stress and used multivariate statistical analysis to realize the optimal period of yield estimation. It is well reported that yield loss caused by frost generally depends on the intensity of the low temperature and its duration^35^. Hence, in this study, field and pot experiments were performed in which winter wheat was treated with different low-temperature stresses with different time during the jointing stage. Based on the canopy hyperspectral data and yield of frozen winter wheat measured at different growth stages, the relationship between the hyperspectral data and winter wheat freeze stress was determined by statistical methods that could extract the hyperspectral wavelength information vital for estimating winter wheat production. In addition, a hyperspectral estimation model was established to achieve early estimation of winter wheat yield under frost damage. Therefore, 3 different aims were designed in this study, 1. to analyse the response of winter wheat canopy spectra, yield and yield components at different stages; 2. to extract sensitive bands by the SPA; and 3. to analyse the optimal period for estimating production after modelling with the SPA-MLR method.

## Results and Analysis

### Effect of freeze stress on growth status of winter wheat

**Figure 1:**
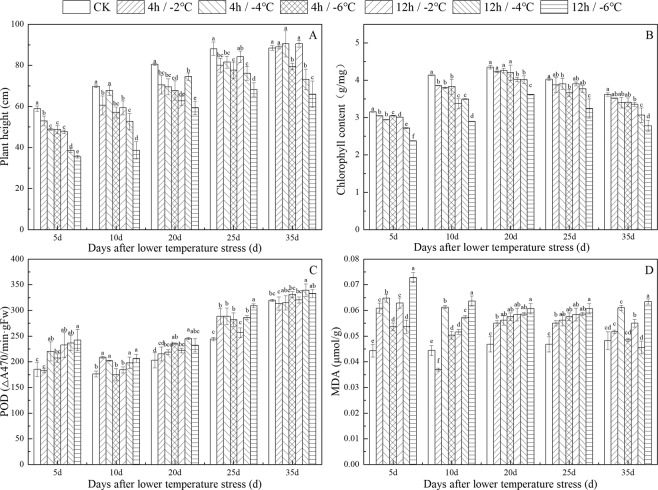
Influence of different freezing treatments on Plant height (**A**), Chlorophyll (**B**), POD (**C**) and MDA (**D**). Different small letters refers meant significant difference among treatments at 0. 05 level.

Plant height, an important character of winter wheat, has very important influence on its yield. The height of winter wheat increased gradually with the advancing of growth stage. The height of winter wheat in different treatments but at the same stage showed the same pattern, in which the height decreased gradually with an increase in freezing degree. On 35 days after freeze stress, the height of winter wheat treated by freezing at 4 h/−6 °C, 12 h/−4 °C and 12 h/−6 °C were still significantly lower than the CK, but the difference between the other treatments and the CK was not obvious. It indicated that winter wheat had the ability of self-repair in a certain extent of freeze stress (Fig. 1A).

Green plants use light energy for photosynthesis by chlorophyll, the amount of which will directly affect crop yield. The chlorophyll content of winter wheat first increased and then decreased after different freeze stress treatments, and the chlorophyll content reached a maximum at 20 days after freeze stress. Thereafter, the nutrients in the leaves began to transfer to the ear, and the chlorophyll content continued to decrease. The chlorophyll content of winter wheat in different treatments but at the same stage showed the same pattern, in which the chlorophyll content decreased gradually with an increase in freezing degree (Fig. 1B).

Upon plant exposure to freeze stress, peroxidase (POD) have a defensive effect, and work interactively to scavenge excess free radicals in order to maintain a normal dynamic level of free radicals in the plant, which could improve the ability of plants to resist stress and reduce the plant damage caused by adversity. The POD activity with an increase in the number of days after freeze stress. POD activity was higher after freezing treatment, indicating that POD was sensitive to freeze stress and was the main protective enzyme allowing winter wheat to adapt to low-temperature stress (Fig. 1C). Malondialdehyde (MDA) is one of the main products of lipid peroxidation, the level of which directly reflects the degree of lipid oxidation. Therefore, the degree of plant damage could be understood by measuring the changes in MDA content. The MDA content of winter wheat under different frost damage treatments increased little with an increase in the number of days after freeze stress. The MDA content in the winter wheat increased gradually with the increase in the degree of freeze stress at 20 days and 25 days after freeze stress, the difference in which was significant (Fig. 1D).

### Effect of freeze stress on grain yield

**Figure 2:**
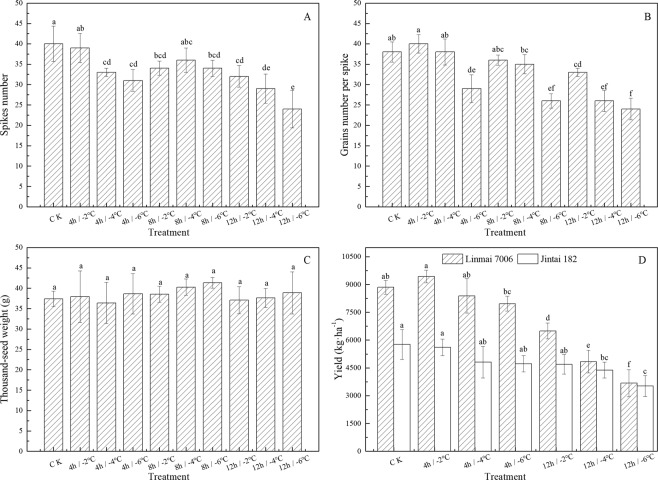
Effect of lower temperature stress on Spikes number (**A**), Grains number per spike (**B**), Thousand-seed weight (**C**) and Yield of different varieties winter wheat (**D**).

Figure 2 shows the changes in the Spikes number(SN), Grains number per spikes(GNPS) and thousand-grain weight(TGW) of Linmai 7006 after freeze stress.When the treatment duration was 4 h or 12 h, the PN decreased as the temperature decreased. When the treatment temperature was −2 °C, the PN showed a decreasing trend. Under the treatment of 12 h/−6 °C, the PN was the smallest, at 24 spikes, which was 12 spikes fewer than observed in the CCK, declining by 33.33% (Fig. 2A). There were only 40 and 38 grains under 4 h/−2 °C and 4 h/−4 °C, respectively. The GNPS under the other treatments was also lower than that of the CCK; For the same low-temperature treatment duration (8 h or 12 h), The GNPS decreased with a gradual decrease in temperature. When the treatment duration was 8 h and the treatment temperature decreased from −4 °C to −6 °C, the GNPS decreased from 35 to 26, which was the largest decrease, indicating that the treatment temperature had a stronger influence on the GNPS (Fig. 2B). The TGW did not change significantly. With an increase in stress duration at the same treatment temperature, the TGW first increased and then decreased, but the changes were minor (Fig. 2C). After freeze stress, the yield of two varieties winter wheat decreased to different extents that the Linmai 7006 is higher than Jintai 182 (Fig. 2D). Only the Linmai7006 treatments of 4 h/−2 °C did not produce this result, and there was no reduction in production. When the treatment temperature was −4 °C, with increasing treatment duration (i.e., treatment for 4 h to 12 h), the yield of Linmai 7006 decreased from 8387.4668 kg·ha^−1^ to 4840.432 kg·ha^−1^, respectively, which were lower than the value in the CK of 8852.20 kg·ha^−1^, indicating that under the same temperature condition, the yield decreased with increasing freeze stress duration. When the treatment time was 12 h, the Linmai7006’s yields at −2 °C, −4 °C and −6 °C were 6495.64 kg·ha^−1^, 4840.43 kg·ha^−1^ and 3677.54 kg·ha^−1^, respectively, showing that with the decrease in stress temperature, the yield decreased at the same treatment duration. After freeze stress, the yield of Linmai 7006 decreased more than that of Jintai 182, which indicated that Jintai 182 had higher resistance to low temperature stress.

### Descriptive analysis of grain yield data

The yield data from the pot experiment (2015) were taken as a calibration set with 20 samples, and two years’ yield data (2015 and 2016) from the field experiment were used as a validation set with 14 samples. Descriptive statistics of the calibration set and validation set of yield data are shown in Table 1.

**Table 1:** Descriptive statistical analysis for the yield of winter wheat.

Variables	Data set	Sample number	Range	Min	Max	Average	SD	Skewness	Kurtosis
Yield (kg·ha^−1^)	Calibration set	20	6622.970	2810.680	9433.640	6093.200	2065.570	0.252	−1.181
	Validation set	14	6428.33	2423.87	8852.200	4793.750	1696.860	0.895	1.182

Table 1 shows that the winter wheat yield in the pot and field tests was 6622.970 kg·ha^−1^ and 6428.330 kg·ha^−1^, respectively, the SDs of which were 2065.570 kg·ha^−1^ and 1696.860 kg·ha^−1^. At the same time, the calibration and validation sets’ maximum values were 9433.200 kg·ha^−1^ and 8852.200 kg·ha^−1^, minimal values were 2810.680 kg·ha^−1^ and 2423.870 kg·ha^−1^, and averages were 6093.200 kg·ha^−1^ and 4793.750 kg·ha^−1^, respectively. The results showed that the yield in the pot experiment was greater than that in the field experiment. Because the skewness (0.895) in the validation set followed 0 < skewness < 1, the distribution was highly skewed to the right. From a statistical point of view, the data in the calibration set and validation set met the statistical assumptions required for further analysis of the winter wheat freeze stress treatment; therefore, data analysis could be carried out.

### Response of canopy spectra on freeze stress

**Figure 3:**
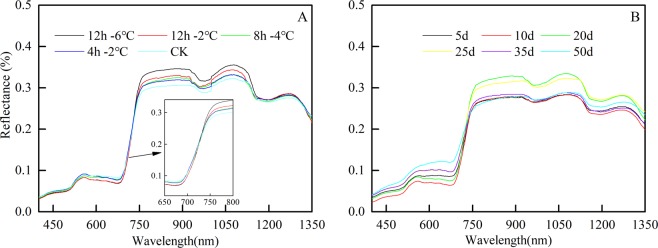
Response of the canopy spectrum of winter wheat to low temperature stress. (**A**) the spectral reflectance of different freeze stress treatments at the same growth stage (**B**) the reflectance of different growth stages of the same freeze stress treatment.

The spectral reflectance curves obtained 5 days after freeze stress had the same spectral signatures, but there was a moderate difference in reflectivity (Fig. 3A). The spectral reflectance of the control group of calibration(CCK) was highest in the visible region, and the reflectance gradually decreased with an increase in the degree of frost stress. However, the spectral reflectance of the CCK was the lowest in the near-infrared band and increased with the degree of freeze stress. The spectral reflectance in the red edge region gradually decreased with an increase in the degree of frost stress. The red edge position showed a shift towards short wavelengths (blueshift phenomenon). Except at fifty days after freeze stress, the changes in the canopy spectral curves in the rest of the reproductive stages remained the same, and at 20 days, the spectral reflectance was high in the near-infrared band (Fig. 3B) under the same low-temperature stress treatment (−2 °C/12 h). The red edge position exhibited a “blueshift” in the red edge area. The physiological changes and morphological changes in frozen winter wheat revealed a decrease in plant height and chlorophyll content with an increase in POD and MDA in the plant after low-temperature injury. These results proved that the canopy spectrum was sensitive to freeze stress.

### Correlation analysis

**Figure 4:**
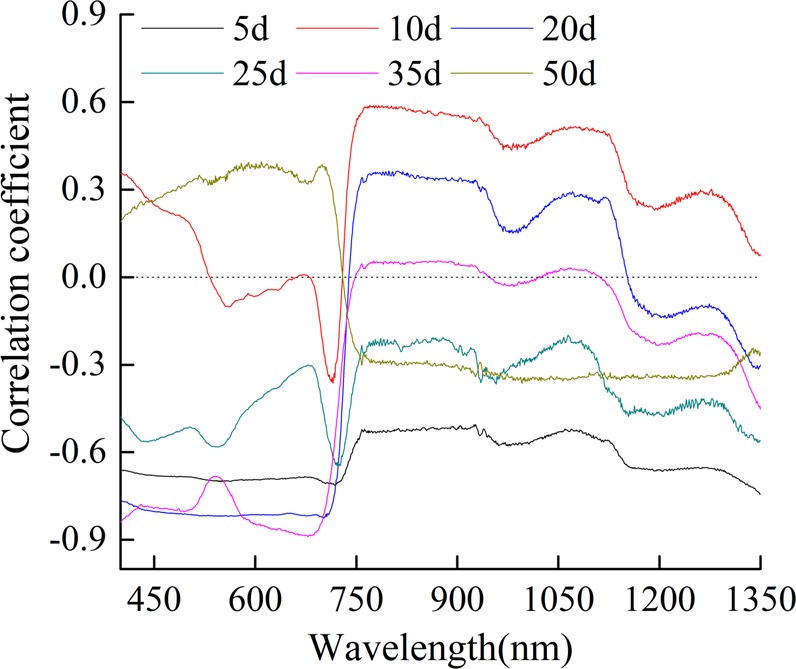
Correlation coefficient between yield of winter wheat and canopy spectrum on different days after freeze stress.

It can be seen from Fig. 4 that there was a moderate correlation between grain yield and the spectrum. The negative correlation reached approximately −0.8 in the range of 476–490 nm in the blue light band at 20 days and 35 days after freeze stress. The best correlation was −0.65, which occurred at approximately 550 nm at 35 days after freeze stress. The correlation coefficient reached −0.9 at 680 nm. In conclusion, freeze stress of the winter wheat canopy and the yield had a good correlation, which indicated the feasibility of monitoring crop yield.

### Spectral region analysis

**Figure 5:**
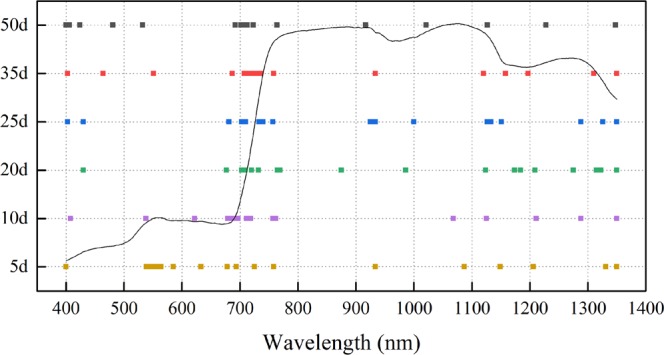
Important spectral regions under the lower temperature stress at different stages.

The SPA was used to select the important spectral region for the yield of winter wheat on different days after low-temperature stress, and the results are presented in Fig. 5.

Figure 5 shows that approximately 38% of the extracted important variables were located in the red edge characteristic spectral region (680 nm–780 nm), as we can see that the specific information contained in the red edge region was very concentrated. For example, at 10 days after freeze stress (687 nm, 708 nm, 715 nm, 721 nm, 722 nm, 723 nm, 732 nm, 736 nm and 758 nm) and at 25 days (703 nm, 709 nm, 720 nm, 732 nm, 765 nm, 769 nm). About 50% extracted important bandswere in the near-infrared region (925 nm, 934 nm, 1000 nm, 1127 nm, 1133 nm, 1151 nm, 1288 nm, 1326 nm and 1350 nm) at 20 days after freeze stress, and a total of 56% of the extracted important bands were in the near-infrared region (875 nm, 986 nm, 1124 nm, 1174 nm, 1184 nm, 1209 nm, 1275 nm, 1315 nm, 1323 nm and 1350 nm) at 25 days after freeze stress. From a physiological point of view, there would be obvious physiological and biochemical changes in winter wheat plants after low-temperature injury, such as a decrease in chlorophyll and enzyme activities, which would ultimately influence the growth of winter wheat. Many studies have confirmed that the red edge region and the near-infrared region of the canopy spectrum are sensitive to crop growth status after stress treatment. The red edge region and the near infrared region were closely related to the growth of winter wheat after the low-temperature treatment.

### Spectral monitoring models of winter wheat yield

Three models were established, namely, a whole spectrum predictive model based on principle component regression (PCR), an NDVI model based on multiple linear regression (MLR) and an SPA-MLR model, respectively. The results are shown in Table 2. The model evaluation coefficient (R^2^) of the model established based on the NDVI was lower than that of the models established by PCR and SPA-MLR. The yield estimation calibration model established based on the whole-spectrum predictive models (R^2^ = 0.879, RMSEC = 700.921, RPD = 2.872) and NDVI (R^2^ = 0.791, RMSEC = 847.378, RPD = 1.945) at 25 days after freeze stress showed the best performance, and the RPD values of the NDVI models were below 1.4 at 5, 10 and 50 days after freeze stress, The validation model established based on the whole-spectrum predictive models (R^2^ = 0.854, RMSEP = 625.700, RPD = 2.364) at 25 days after freeze stress had the strongest stability, but the accuracy of validation model established based on NDVI (R^2^ = 0.731, RMSEP = 1565.103, RPD = 0.235) at 25 days after freeze stress was the lowest, indicating that the prediction ability of the model was low and the model was not suitable for practical application (Table 2). Based on the extracted spectral variables, multiple linear regressions (MLRs) were applied to construct a predictive model of winter wheat yield at different growth stages, and a field experiment was used to validate model performance.

**Table 2:** Models performance of winter wheat yield based on different variable at different growth stages.

Variable	Modeling method	Days after lower temperature stress	Calibration set			Validation set		
			R^2^	RMSEC	RPD	R^2^	RMSEP	RPD
Full spectrum	PCR	5d	0.774	956.924	2.104	0.596	1938.589	1.260
		10d	0.743	1020.769	1.972	0.306	1445.399	0.496
		20d	0.777	951.373	2.116	0.718	1083.506	0.684
		25d	0.879	700.921	2.872	0.854	625.700	2.364
		35d	0.815	866.436	2.324	0.742	833.556	1.700
		50d	0.828	835.569	2.409	0.769	967.149	1.766
NDVI	MLR	5d	0.619	1144.250	1.274	0.527	1904.611	0.545
		10d	0.662	1077.768	1.399	0.539	1961.395	0.747
		20d	0.734	955.582	1.661	0.670	1298.490	0.577
		25d	0.791	847.378	1.945	0.731	1565.103	0.235
		35d	0.691	1030.807	1.494	0.760	933.235	1.585
		50d	0.577	1204.692	1.169	0.682	2884.032	1.003
Spectral characteristic variable	SPA-MLR	5d	0.814	1115.045	2.199	0.549	1894.386	1.039
		10d	0.869	765.267	2.751	0.725	1003.529	1.293
		20d	0.874	807.254	2.779	0.745	1047.131	0.840
		25d	0.887	716.985	2.719	0.841	835.060	2.087
		35d	0.787	1073.952	2.099	0.823	768.487	1.487
		50d	0.714	1408.501	1.670	0.676	1041.161	1.647

It was shown that the SPA-MLR models performed best in estimating yield when the growth time was 25 days after freeze injury (R^2^ = 0.887, RMSEC = 716.985, RPD = 2.719; R^2^ = 0.841, RMSEP = 835.060, RPD = 2.087). The model performance at 5 days, 10 days, 20 days and 35 days was lower than that recorded at 25 days after freeze stress. At 50 days after freeze stress, the accuracy and robustness of the calibration model (R^2^ = 0.714, RMSEC = 1408.501, RPD = 1.670) were the lowest, the fitting degree of the validation model (R^2^ = 0.676, RMSEP = 1041.161, RPD = 1.647) was the worst. Furthermore, with the advancement of the birth process, the model coefficient of determination increased and then decreased, the maximum was observed at 25 days after freeze stress.

## Discussion

Accurate and timely crop yield forecasts are critical for developing informed agricultural policies and investments, as well as increasing market efficiency and stability^36^. Climate-smart management is still needed to further improve yields in the wheat-growing regions of China^37^. Moreover, it is well cited that both high and low temperature directly affect the growth and development of winter wheat^38–41^. In addition, low-temperature, low-temperature duration and the interaction of low-temperature level and duration had significant negative effects on wheat physiological index, biochemical index and grain yield^42^. Under different temperatures and duration of freeze stress, winter wheat cells lose water, and cell sap freezes, which is accompanied by plasmolysis, leading to dehydration and solidification of the protoplasm, the loss of vitality, and freezing^43^. Under freeze stress, the canopy leaves of winter wheat will turn yellow and wilt to different degrees, which may be due to the decreases in photosynthesis and chlorophyll content after freeze stress, resulting in the yellowing of leaves. As canopy structure changes, canopy spectral reflectance in the near infrared region also changes^16,17^. Huang *et al*. Reported that the response of the red edge parameter was sensitive to freeze stress and a “blueshift” of the red edge occurred. The green peak decreased, and the red valley gradually changed to the level in the visible spectrum^44^. With an increase in the severity of low-temperature injury, the spectral reflectance frequency increased obviously, and the position of the red edge moved towards the short wavelengths. With an advancement in the growth process, the “green peak” of the visible light band at approximately 550 nm decreased, and the 680 nm red valley increased. The reflectance in the visible light band decreased gradually. It was proven that the spectrum of the winter wheat canopy was sensitive to the low-temperature injury which is feasible to monitor by spectral technology.

The correlation coefficient between winter wheat yield and canopy spectral characteristics was extreme in the red edge area, and this kind of spectral variable is often regarded as a sensitive and characteristic spectral region^45^. The red edge position was closely related to crop growth, which is commonly used to characterize the crop growth status^46–49^. The specific spectral information is obviously concentrated in the red edge region, which showed that the red edge region contains some important information on winter wheat growth after freeze stress.

The yield estimation model established by SPA-MLR was superior to the yield estimation model established based on the NDVI. The reason for this difference may be attributed due to that the SPA-MLR band was more selective and suitable for winter wheat yield estimation after freeze stress. Although the model based on the full spectrum also performed well, the model based on SPA-MLR was more rapid and convenient. The estimation model of frozen winter wheat yield based on SPA-MLR was found to perform best at 25 days after freeze stress and worst at 50 days, which could be due to the reason that a series of physiological and biochemical reactions occurred in the winter wheat plants at the jointing stage, which affected normal growth in a short period of time. However, plants exhibit their own recovery and anti-stress functions up to a certain degree of stress so that they can avoid and alleviate injury^50,51^. Therefore, the severity of the low-temperature injury had an effect on the recovery of plants in a short period of time and determined the growth or yield of winter wheat, and yield estimation was most accurate at 25 days after the freeze stress. The performance at 5, 10, 20 and 35 days after freeze stress was lower. The winter wheat yield estimation at 50 days was observed to be the least. At this time, the winter wheat was in the late growth stage, and the relationship between growth and yield formation was weak. The results revealed leaf injury, leaf wilting and a decrease in chlorophyll content in the short term after freeze stress. With time, the wheat exhibited some resistance to stress and found to be recovered within 35 days after being frozen. At 25 days after stress, the monitoring of winter wheat growth and the prediction of yield were effective, which could help the government departments of agriculture and agricultural insurance develop relevant disaster prevention measures to avoid the loss of winter wheat yield after stress.

We realized the yield estimation of winter wheat after freeze stress in our study. However, there are certain limitations in the yield estimation only based on the empirical method of SPA-MLR as the lower validated model performance. At present, many more physically-based models have wider applicability as it can simulate the processes growth and development of crop, while the technology we used just instantaneously monitor some growth and development of winter wheat, which may limit the validated model performance in our study. Thus, only using one method may be insufficient to obtain high prediction on the yield of winter wheat. In the future research, combining the hyperspectral technology based on the empirical method and the physically-based models may be useful and applicable in improving the grain yield estimation suffered from the low temperature.

## Conclusion

This study aims at the response of the canopy spectrum of winter wheat under freeze stress by artificial low-temperature stress treatments at the jointing stage. Early estimation of yield was achieved. After freeze stress, the canopy spectrum was sensitive to changes in winter wheat, and the red edge region was closely related to the growth condition and yield. The yield estimation model established 25 days after freeze stress had the best performance (R^2^ = 0.887, RMSEC = 716.985, RPD = 2.719), and the prediction of the verified model was accurate (R^2^ = 0.841, RMSEP = 835.060, RPD = 2.087), with strong stability. Based on our results, it was concluded that the real-time monitoring and yield estimation of winter wheat by spectral technology are feasible and effective.

## Materials and Methods

### Experimental design

The experiment was carried out from September 2014 to June 2016 at the Institute of Dry Farming Engineering, Shanxi Agricultural University (N 37°25’, E 112°33’). In this study, two experiments were conducted, one in pots and one in the field. The pot test (2015) was mainly used to extract spectral characteristic information and establish an estimation model. The field tests (2015 and 2016) was used to verify the accuracy of the spectral characteristic information and estimate the performance of the model. The winter wheat varieties used in the tests were Jintai 182 and Linmai 7006, respectively, and the soil used was calcareous cinnamon soil developed from loess parent materials. Jintai 182 is a strong winterness wheat variety, and Linmai 7006 is a weak winterness wheat variety. The soil alkaline nitrogen content was 53.82 mg·kg^−1^, the effective phosphorus content was 18.44 mg·kg^−1^, the rapidly available potassium content was 236.91 mg·kg^−1^, and the organic matter content was 22.01 g·kg^−1^. The same seeding density was used in both experiments. The soil fertility was moderate, and the experimental soil in the pots was from the field. According to the planting density requirements in the Jinzhong area, the planting density in all pots and the field experiment was 6 × 10^6^ plants·ha^−1^.

#### Pot test

The pot specifications were as follows: external diameter: 22 cm, and height: 20 cm. First, 40 seeds were sown in each pot, and seedlings were grown to the three-leaf stage, at which point they were thinned to 20 plants with uniform growth. Second, we applied 80 g of organic fertilizer and 0.5-cm-thick sand (to prevent the soil from hardening) in each pot. Third, the pots were buried in the field and managed normally, until they were taken out of the soil during the winter wheat jointing stage (April 15 to 16, 2015). Fourth, the freeze stress treatment was carried using a plant growth chamber (model: 3760; Thermo Fisher Scientific Inc., Marietta, USA). The temperature treatments were −2 °C, −4 °C, and −6 °C, and the durations were 4 h, 8 h, and 12 h. There were 9 treatments in total. At the end of the treatments, we placed the pots in the field and managed them with normal field practices (Table 3).

**Table 3:** Settings of low-temperature stress treatments.

Data set	Representations	Treatments	
		Time (hours)	Temperature (°C)
Calibration set	4 h/−2°C	4	−2
	4 h/−4 °C		−4
	4 h/−6 °C		−6
	8 h/−2 °C	8	−2
	8 h/−4 °C		−4
	8 h/−6 °C		−6
	12 h/−2 °C	12	−2
	12 h/−4 °C		−4
	12 h/−6 °C		−6
	CCK		
Validation set	4 h/−2 °C	4	−2
	4 h/−4 °C		−4
	4 h/−6 °C		−6
	12 h/−2 °C	12	−2
	12 h/−4 °C		−4
	12 h/−6 °C		−6
	VCK		

#### Field test

We selected 1 m^2^ of homogeneous land in the field to carry out freeze stress treatment (2015 and 2016), by a homemade field freezing test box (internal specifications: 27 cm × 25 cm × 30 cm, lowest possible temperature: −10 °C). We applied the same freeze stress treatment as in the pot test (Table 3).

### Measurement of canopy spectral reflectance

Canopy spectral reflectance was measured by a Field Spec3 portable ground spectrometer (ASD Company, USA). The measured band range was 350–2500 nm, and the field of view was 25°. The spectral data were collected from days five to fifty with an interval of five days after freeze stress, and the determination time was between 10:00 and 14:00. While measuring, the sensor probe was vertically downward, and the whiteboard was corrected in each measurement. For the wheat canopy, the test distance of the pots was 30 cm, while that in the field test was 1 m. Each measurement recorded 8 lines of the spectral reflectance, obtained the average value and finally derived the data.

### Measurement of the yield index

Three pots of winter wheat with uniform growth were selected from each treatment during the harvest period, and their yield components, such as Spikes number, Grains number per spike, and Thousand-grain weight, were measured. In the field experiment, three 1 m^2^ of winter wheat with the same growth and uniform management was selected as the sample area for each treatment. Then, the yield components were measured, and the units were converted to kg·ha^−1^.

### Data processing and analysis

We preprocessed the spectral data and selected 400–1350 nm for study because it is the band of absorption for water vapor, which influences reflectivity. After this process, we used MATLAB software to conduct correlation analysis, successive projections algorithm (SPA) analysis and multiple linear regression (MLR).

### Model evaluation

The coefficient of determination (R^2^) was used to represent the fitting degree of the predicted values and the measured values. Root-mean-square error (RMSE) was used to represent the predictive ability of the model, and predictive residuals (RPD) were used to represent the stability of the models. Chang *et al*. found that when RPD > 2, the model had better prediction ability; when the RPD value was between 1.4 and 2, the prediction ability of the model was general; and when RPD < 1.4, the prediction ability of the model was poor, indicating that the model was not suitable for prediction^52^. The validation parameters (R^2^, RMSE, and RPD) were calculated as follows:

1 $${R}^{2}=1-\frac{{\sum }_{i=1}^{n}({Y}_{i}-{Y^{\prime} }_{i})/(n-p-1)}{{\sum }_{i=1}^{n}{({Y}_{i}-{Y^{\prime} }_{i})}^{2}/(n-1)}$$

2 $$RMSE=\sqrt{\frac{1}{n}{\sum }_{i=1}^{n}{({Y}_{i}-{Y^{\prime} }_{i})}^{2}}$$

3 $$RPD=\frac{SD}{RMSE}$$
